# Desmitis of the palmar or plantar ligaments of the proximal interphalangeal joint: A descriptive case series

**DOI:** 10.1111/evj.14547

**Published:** 2025-06-12

**Authors:** Manon W. J. Peeters, Stephan Ott, Elisabeth van Veggel, Dagmar Berner, Melanie Perrier

**Affiliations:** ^1^ Department of Clinical Science and Services Royal Veterinary College Hatfield UK; ^2^ Pferdeklinik am Kottenforst GmbH Wachtberg‐Villiprott Germany; ^3^ Sporthorse Medical Diagnostic Centre Heesch The Netherlands

**Keywords:** advanced imaging, desmitis, horse, lameness, pastern

## Abstract

**Background:**

Limited reports in the literature are available regarding desmitis of the palmar/plantar ligaments of the proximal interphalangeal joint (PL‐PIPJ); the clinical significance of such injuries is unknown.

**Objectives:**

To describe the ability to differentiate the PL‐PIPJ on low‐field magnetic resonance (MR) examination. To describe the injury characteristics of the PL‐PIPJ on MR examination and the correlation with clinical features and lameness.

**Study Design:**

Retrospective case series.

**Methods:**

Data and MR images of 29 horses were collected from the databases of three institutions. Horses were included when desmitis of the PL‐PIPJ was present, specifically the axial palmar/plantar ligament, the abaxial palmar/plantar ligament, the proximal enthesis of the distal digital annular ligament, and the distal enthesis of the proximal digital annular ligament. The clinical features and lameness scores were noted for all cases. The MR examinations were reviewed. Ligaments were graded for visibility and degree of pathology by a board‐certified diagnostic imaging specialist.

**Results:**

Differentiation between the different ligaments is not always reliably possible, especially as the abaxial palmar/plantar ligament was only distinctly visible in 4 out of 80 ligaments (5%, 95% confidence interval (CI): 1%–12%). Desmitis of the PL‐PIPJ was the main MR finding in 13 out of 29 (45%, CI: 26%–64%) clinical cases. Periligamentous oedema was evident in 53 of the 71 (74%, CI: 63%–84%) abaxially located ligaments with desmitis. Enthesophyte formation was present in 34 out of 102 ligaments with desmitis (33%, CI: 24%–43%).

**Main Limitations:**

Retrospective nature of the study, lack of control.

**Conclusions:**

Pathology to the PL‐PIPJ can be a primary cause of lameness in horses and these structures should therefore be critically evaluated on MR examination. Differentiation between the abaxially located structures can be challenging. Periligamentous oedema is often present in cases of desmitis of the abaxially located ligaments; its presence should prompt closer assessment.

## 1 INTRODUCTION

**FIGURE 1:**
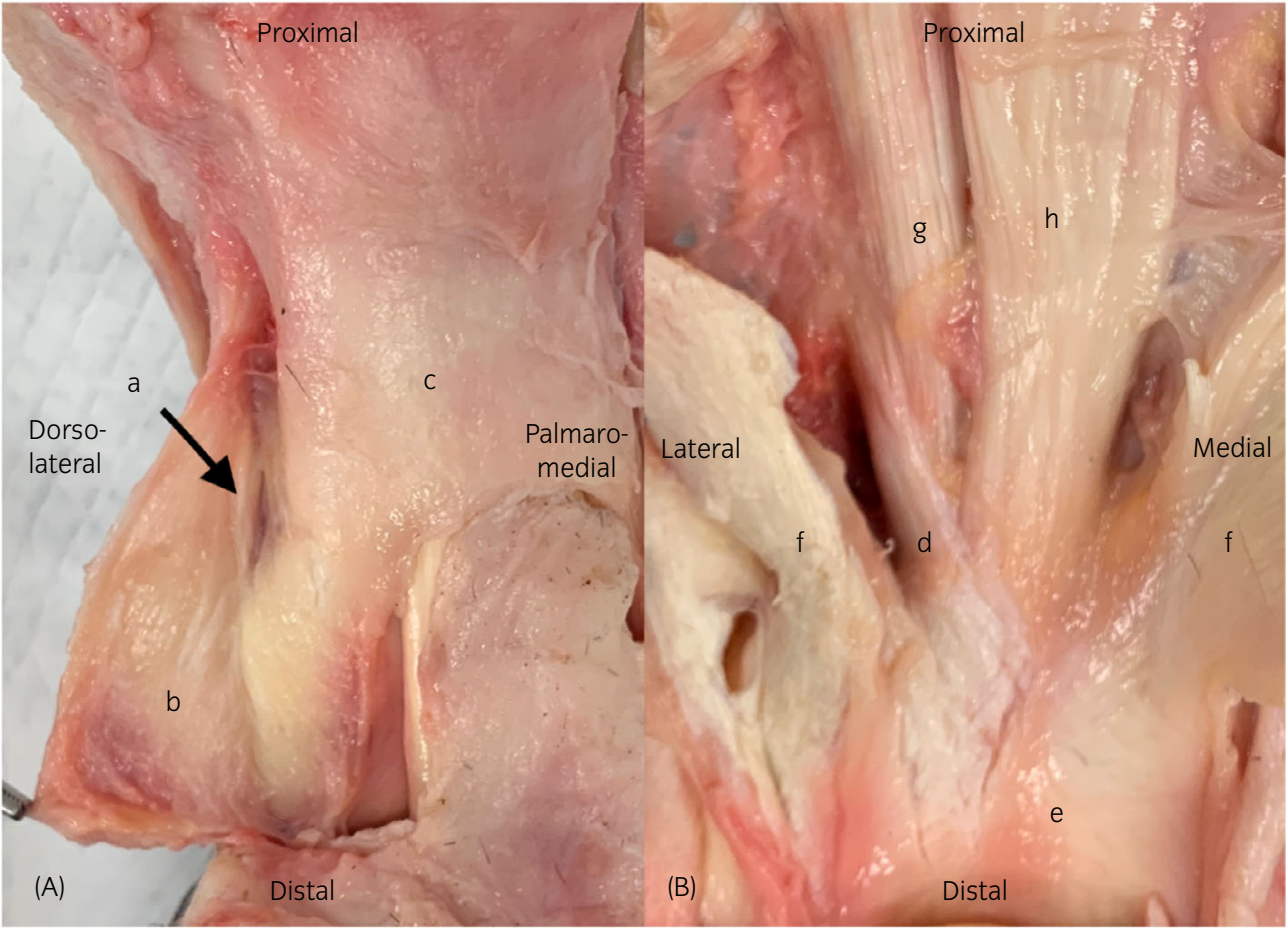
Anatomical specimens giving an impression of the location of the ligaments. Picture (A) highlights the palmarolateral aspect of the pastern region. The abaxial palmar ligament (a) is located axially to the distal digital annular ligament (b) and abaxially to the proximal digital annular ligament (c). Picture (B) shows the palmar aspect of the pastern after removal of both digital annular ligaments and the deep digital flexor tendon. The axial palmar ligament (d) attaches onto the middle scutum (e), is located axially to the superficial digital flexor tendon branch (f) and distoabaxially to the oblique distal sesamoidean ligament (g). The straight distal sesamoidean ligament (h) is indicated for reference.

There are several ligaments at the level of the palmar/plantar aspect of the proximal interphalangeal joint. The abaxial palmar/plantar ligaments (ABPLs) are located proximopalmar/plantar to the collateral ligaments of the proximal interphalangeal joint.^1^ The axial palmar/plantar ligaments (APLs) originate distal to the insertion of the oblique sesamoidean ligaments on the proximal phalanx (Figure 1).^2^ Both the ABPL and APL form part of the middle scutum.^3^ The APL originates axial to the superficial digital flexor tendon branch and abaxial to the straight sesamoidean ligament (Figure 1).^4^ The ABPL originates abaxial to the distal insertion of the PDAL and axial to the proximal origin of the DDAL (Figure 1).^2^ The proximal digital annular ligament (PDAL) originates at the proximolateral and proximomedial aspect of the proximal phalanx and inserts on the distolateral and distomedial aspect of the proximal phalanx. The distal digital annular ligament (DDAL) originates from the medial and lateral distal aspect of the proximal phalanx and inserts on the distal phalanx along with the deep digital flexor tendon.^5^ The origin of the DDAL is located abaxial to the insertion of the PDAL, separated by the ABPL (Figure 1).^2^ The proximal interphalangeal joint has a low range of motion, approximating 30 degrees and consisting primarily of flexion and extension.^3^, ^6^ The APL and ABPL prevent hyperextension of the proximal interphalangeal joint together with the straight sesamoidean ligament.^3^

Injuries to the palmar/plantar ligaments of the proximal interphalangeal joint (PL‐PIPJ) are an uncommonly reported cause of lameness in the horse.^4^ Primary injuries to the DDAL have been described in a case series including seven horses, where thickening of the DDAL was noted on high‐field magnetic resonance (MR) examination.^5^ Reported injuries to the PDAL are thickening and avulsion fractures at the level of its origin.^7^, ^8^ However, no reports on the injury of the insertion have been published.

**FIGURE 2:**
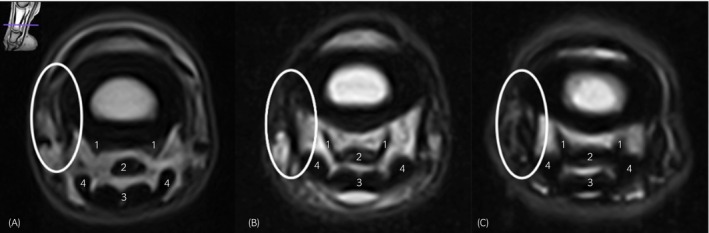
T2W FSE image at the level of the proximal phalanx, lateral is to the left, obtained using the 0.25 Tesla magnetic resonance system under general anaesthesia. Illustrating the normal appearance of the palmar ligaments proximal (A), mid (B) and prior to insertion on middle scutum (C). Number 1 indicates the axial palmar ligaments. The white oval shape illustrates the difficulty in differentiating the abaxially located structures (abaxial palmar ligament, proximal enthesis of the distal digital annular ligament and distal enthesis of the proximal digital annular ligament). The other numbers are 2: Straight distal sesamoidean ligament; 3: Deep digital flexor tendon; 4: Superficial digital flexor tendon branch.

The ligaments are very small and are closely opposed to one another (Figure 2); low‐field MR examination utilises slice thicknesses (2–6 mm) which may exceed the normal thickness (<2 mm) of these ligaments, thus it is difficult to reliably differentiate these structures and potentially therefore to identify changes to these structures on low‐field MR examination. Despite this, MR examination is considered by many as the gold standard for diagnosing soft tissue injuries in the pastern and foot.

The ligaments included in this study are the ABPL, the APL, the distal enthesis of the PDAL and the proximal enthesis of the DDAL. The aim of this study was to describe the clinical and diagnostic imaging findings of horses diagnosed with an associated injury of these ligaments on low‐field MR examination. The objectives of this retrospective study were to:

1. Describe the ability to differentiate the individual PL‐PIPJ.
2. Describe the injury characteristics of the PL‐PIPJ on low‐field MR examination.
3. Describe any relationships between the pathology of the PL‐PIPJ, clinical features, and lameness.

## 2 MATERIALS AND METHODS

**FIGURE 3:**
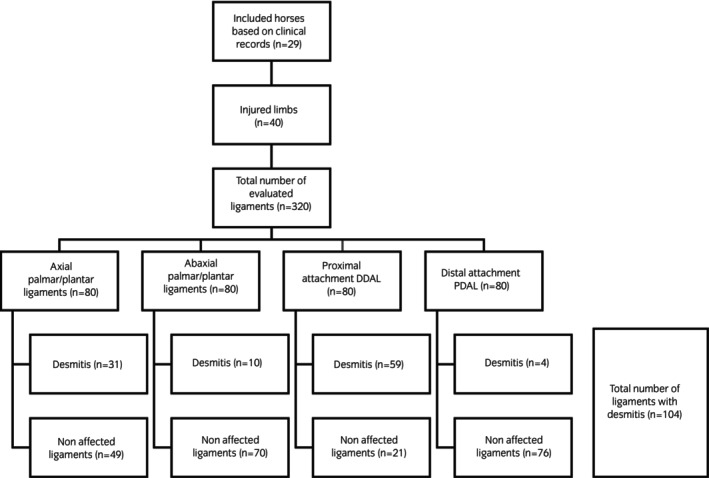
Flowchart illustrating the number of injured limbs, evaluated ligaments, and the differentiation in between the different subcategories. DDAL, distal digital annular ligament; PDAL, proximal digital annular ligament.

A retrospective case series was conducted in collaboration between three institutions: Equine Referral Hospital of the Royal Veterinary College (RVC), United Kingdom, Pferdeklinik am Kottenforst GmbH (KF), Germany, and Sportshorse Medical Diagnostic Centre (SMDC), The Netherlands. Clinical records were searched for horses that underwent low‐field MR examination of the foot or pastern, with pathology to structures located at the palmar or plantar aspect of the proximal interphalangeal joint. Horses were included (Figure 3) if signs of desmitis were identified on low‐field MR examination to either an individual or a combination of the following ligaments: ABPL, APL, distal enthesis of the PDAL and proximal enthesis of the DDAL. Signalment, age at time of investigation, relevant history, discipline, and lameness examination findings including response to regional and local anaesthesia were recorded.

### 2.1 Lameness examination

Lameness scores were graded using the American Association of Equine Practitioners' lameness scale (AAEP score); the horses were evaluated using subjective visual evaluation.^9^ Response to diagnostic analgesia was subjectively characterised as mild improvement, substantial improvement, or abolishment of the lameness.

### 2.2 MR examination

MR images of the pastern region were obtained either under general anaesthesia with a 0.25 Tesla (KF) (G‐scan, Esaote) system or in standing horses using a 0.27‐Tesla system (RVC and SMDC) (Equine Limb Scanner; Hallmarq Veterinary Imaging, Ltd) in all cases. Multiple planes and weightings were acquired depending on the clinician's preference. Transverse T2 weighted fast spin echo (FSE) and T1 weighted gradient recall echo (GRE) were obtained in all patients, with short tau inversion recovery (STIR) images obtained in all but one case. Transverse MR images were piloted perpendicular to the proximal phalanx in all cases. The slice thickness used varied for the different sequences, between 1.6 and 5 mm for the T1 GRE, between 4 and 5 mm for the T2 FSE, between 4 and 5 mm for the STIR, and between 3 and 5 mm for the T2* GRE. MR images were uploaded into a free DICOM viewing software (horosproject.org, sponsored by Nimble Co LLC Purview, 2020), reviewed by a board‐certified diagnostic imaging specialist (Dagmar Berner), aware of the inclusion criteria, and graded as shown in Table 1. All ligaments were assessed on the ability to visualise the ligament individually and divided into either ligament not well visualised, ligament visible but with indistinct margins, or ligament visible and clearly delineated from adjacent structures.

**TABLE 1:** Summary of the magnetic resonance examination grading.

Imaging feature	Classification of grading
Location of lesion	Enthesis proximal phalanx
	Mid ligament
	Enthesis middle phalanx
Increased CSA; Increased signal; Periligamentous oedema; Bone oedema‐like signal	Absent
	Mild
	Moderate
	Severe
Lesion type	Diffuse
	Core lesion
Presence of enthesophyte	Absent
	Enthesophyte present proximal phalanx
	Enthesophyte present middle phalanx
Avulsion fractures	Absent
	Present on proximal phalanx
	Present on middle phalanx
Adhesions	Absent
	Present

Abbreviation: CSA, cross‐sectional area.

**FIGURE 4:**
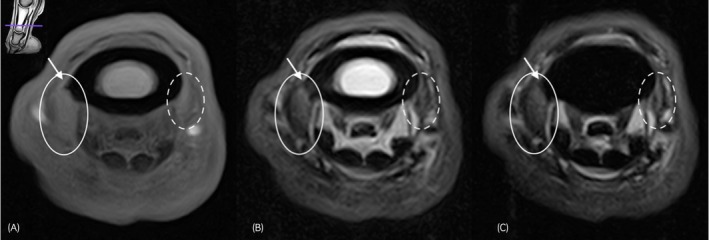
T1W transverse image (A), T2W FSE transverse image (B) and STIR transverse image (C) at the level of the distal aspect of the proximal phalanx, lateral is to the left, obtained using the 0.27 Tesla magnetic resonance system images obtained on a standing horse. There is marked increased cross‐section of the lateral (circle) aspect of distal digital annular ligament (DDAL) and mild increase in cross‐section of the medial aspect of DDAL (dotted circle), both showing mild increase in signal intensity in the T2W FSE and STIR sequences. New bone formation is evident at the lateral origin of the DDAL (arrow). Note that the ligaments are not well visualised in T1W images (A), likely due to magic angle artefact within these ligaments.

The increase in cross‐sectional area was predominantly assessed on the T2 weighted FSE sequences and defined as mild when the ligament was enlarged up to twice the original size, moderate when an enlargement was noted between two and three times the original size, and marked when the size was over three times the normal cross‐sectional area. Figure 4 illustrates the increased cross‐sectional area of the DDAL in a clinical case.

The increase in signal intensity was predominantly assessed on the T2 weighted FSE sequences and defined as mild when a mild increase in signal intensity was noted, moderate when the increase in signal intensity was moderate, and marked when the signal intensity was markedly increased.

A diffuse lesion pattern was characterised as increased signal intensity over the complete cross‐sectional area of the ligament. A lesion was defined as core when the increased signal intensity was focal and within the centre of the ligament.

## 3 RESULTS

### 3.1 Clinical features

Twenty‐nine horses, 17 geldings, and 12 mares with a median age of 11 years, ranging from 4 to 24 years were included in the study. They all had desmitis of either one or a combination of the following ligaments, ABPL, APL, distal enthesis of the PDAL, or proximal enthesis of the DDAL. In 13 cases, the desmitis appeared to be the most predominant MR finding (*n* = 13/29, 45%, 95% confidence interval [CI]: 26%–64%) and in the remaining 16 cases, other structures were found to have potential clinically significant abnormalities. Breeds included Warmblood (*n* = 13/29), Cob (*n* = 7/29), Icelandic horse (*n* = 4/29), Arabian (*n* = 1/29), Irish Draught horse (*n* = 1/29), Pony (*n* = 1/29), and unknown (*n* = 2/29). The horse's discipline was recorded in 23 cases; they were predominantly used for general riding purposes (*n* = 9/23) and jumping (*n* = 7/23) but also showing (*n* = 3/23), dressage (*n* = 2/23) and low‐level eventing (*n* = 1/23).

Lameness grade was recorded in all cases and was located to the forelimbs in 22 cases and the hindlimbs in seven cases. The lameness score using the AAEP grading scale identified a median score of 3 out of 5. The duration of lameness had a median of 6 weeks, ranging from 1 to 308 days. Distal limb flexion tests were performed in 26 cases with a positive response in 21 cases and no response in five cases. Diagnostic anaesthesia was performed in 27 cases. Out of the 13 cases with predominant MR findings on the PL‐PIPJ, 11 underwent diagnostic analgesia. The results of these horses are the following: 6 out of 11 horses had a substantial improvement to palmar/plantar digital nerve block with subsequent abolishment of the lameness to an abaxial sesamoid nerve block; 1 out of 11 horses showed no improvement to the palmar/plantar digital nerve block with abolishment of the lameness to the abaxial sesamoid nerve block. In 3 out of 11 horses, an abaxial sesamoid nerve block was performed without prior localising blocks and resulted in abolishment of lameness; in 1 out of 11 horses, a low four‐point nerve block was performed without prior localising blocks and resulted in abolishment of lameness.

Palpation findings were reported in 11 horses; four horses showed evidence of swelling in the mid pastern region, and five horses showed signs of pain on palpation of the mid pastern region.

### 3.2 MR examination

From the 29 horses included in the study, 11 horses underwent bilateral MR examination. In total, 40 limbs were imaged and found to have abnormalities of either the lateral and/or medial ABPL, APL, distal enthesis of the PDAL, and/or proximal enthesis of the DDAL. Table 2 summarises the appearance of each affected ligament on low‐field MR examination, and Figure 3 further illustrates the number of injured ligaments per category.

**TABLE 2:** Low‐field MR examination results, showing the appearance of the injured ligaments.

	Axial palmar/plantar ligament		Abaxial palmar/plantar ligament		Proximal enthesis DDAL		Distal enthesis PDAL	
	Lateral	Medial	Lateral	Medial	Lateral	Medial	Lateral	Medial
Total number ligaments involved	18	13	4	6	34	25	1	3
Increased cross‐sectional area								
Mild	12	4	1	2	9	14		2
Moderate	6	8	3	4	13	9	1	1
Severe		1			12	2		
Increased signal								
Mild	13	5	2	3	15	18	1	2
Moderate	4	8	2	2	15	4		1
Severe	1			1	4	3		
Location								
Origin	18	13	2	2	34	24		2
Mid ligament			2	4		1	1	1
Insertion								
Lesion type								
Diffuse	18	13	2	6	34	24	1	3
Core			2			1		
Periligamentous oedema								
None	18	13	2	3	7	6		
Mild			1	2	16	16	1	1
Moderate				1	8	1		1
Severe			1		3	2		
Enthesophyte formation								
None	15	13	3	5	15	14	1	2
Mild	1		1	1	11	8		1
Moderate	1				6	1		
Severe					2	2		

Abbreviations: DDAL, distal digital annular ligament; PDAL, proximal digital annular ligament.

The results of the visibility grading of the ligaments are displayed in Table 3. The ABPL was found to be either not visible or visible with indistinct margins in the majority of cases, while only four ligaments were assessable with distinct margins out of 80. The other PL‐PIPJ showed variable visibility; however, their visibility was predominantly good, with or without distinct margins. It was the opinion of the assessor that the presence of desmitis subjectively increased the ability to differentiate the ligaments.

**TABLE 3:** Visibility scoring results.

	Axial palmar/plantar ligament	Abaxial palmar/plantar ligament	Proximal enthesis DDAL	Distal enthesis PDAL
Not well visible	2 /80 (3%, CI: 0%–9%)	17/80 (34%, CI: 24%–45%)	0	7/80 (9%, CI: 4%–17%)
Visible with indistinct margins	13/80 (16%, CI: 9%–26%)	49/80 (61%, CI: 50%–72%)	24/80 (30%, CI: 20%–41%)	27/80 (34%, CI: 24%–45%)
Visible with distinct margins	65/80 (81%, CI: 71%–89%)	4/80 (5%, CI: 1%–12%)	56/80 (70%, CI: 59%–80%)	46/80 (58%, CI: 46%–69%)

Abbreviations: DDAL, distal digital annular ligament; PDAL, proximal digital annular ligament.

**FIGURE 5:**
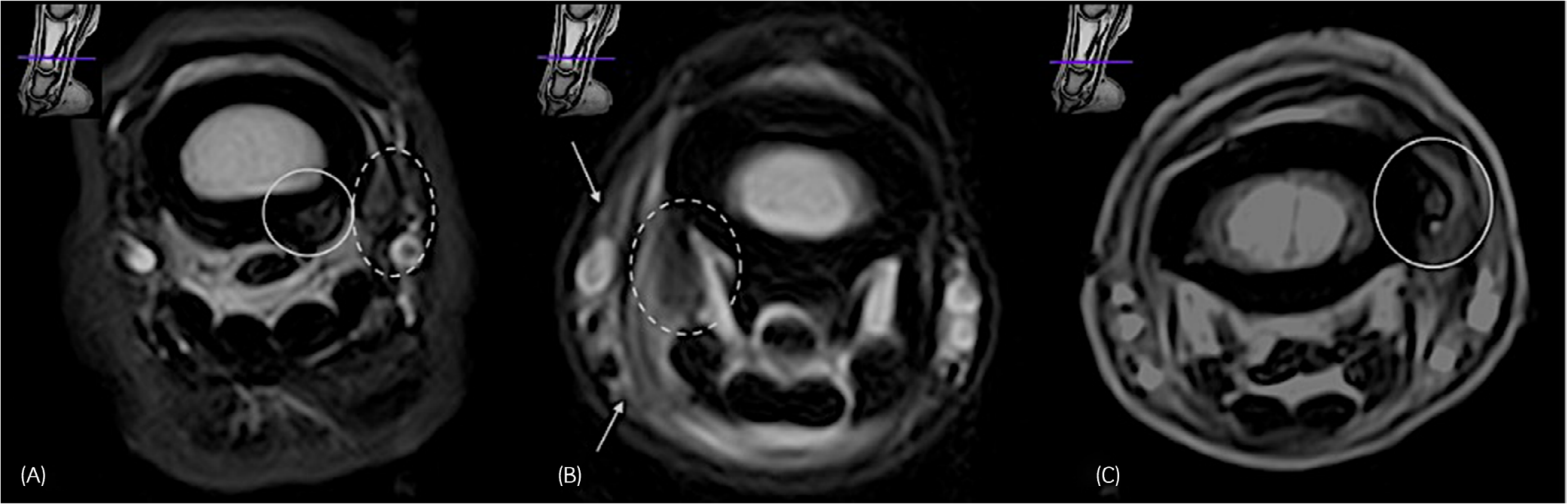
Transverse MR images at the level of the distal aspect of the proximal phalanx. Lateral is to the left of all images. (A) T2W FSE transverse image obtained using the 0.25 Tesla magnetic resonance system under general anaesthesia. There is mild increase in cross‐sectional area and mild increased signal intensity of the axial palmar ligament (circle) and moderate increase in cross‐sectional area and moderate increased signal intensity of the proximal enthesis of the distal digital annular ligament (dotted circle). (B) T2W FSE FAST transverse image obtained using the 0.27 Tesla magnetic resonance system on standing patients. There is desmitis of the abaxial plantar ligament (dotted circle), highlighted by the moderate increased cross‐sectional area and moderate increase in signal intensity with concurrent moderate periligamentous oedema (white arrows). (C) T2W FSE transverse image obtained using the 0.25 Tesla magnetic resonance system under general anaesthesia. This image illustrates the presence of a moderate enthesophyte formation at the insertion of the proximal enthesis of the distal digital annular (circle). The ligament shows a mild increased cross‐sectional area and mild increased signal intensity.

Cases were included in the study based on the presence of both an increased cross‐sectional area and increased signal intensity of the PL‐PIPJ on T2‐FSE weighted low‐field MR examination. Most cases of desmitis had a predominant diffuse lesion pattern, with a core lesion found in two cases with desmitis of the ABPL. Additionally, periligamentous oedema and/or enthesophyte formation were common findings relating to desmitis of the PL‐PIPJ. Periligamentous oedema, illustrated in Figure 5B, was noted in a total of 33 out of the 40 limbs (83%, CI: 67%–93%) and was only evident when pathology to the abaxially located ligaments (Figure 5B) was present. The incidence of periligamentous oedema when desmitis of the abaxially located ligaments was evident was 74% (*n* = 53/71, CI: 63%–84%). Enthesophytes were present in 34 out of 102 ligaments with desmitis (33%, CI: 24%–43%), all located on the proximal phalanx and predominantly correlated with desmitis of the proximal enthesis of the DDAL, as illustrated in Figure 5C. An avulsion fracture at the origin of the proximal enthesis of the DDAL was noted in one ligament with concurrent desmitis of the proximal enthesis of the DDAL. Bone oedema‐like signal was present at the enthesis of 6 out of 102 ligaments (6%, CI: 2%–12%) affected with desmitis in a total of 4 out of 29 horses (14%, CI: 4%–32%). Bone oedema‐like signal was only present in combination with enthesophyte formation (two mild, two moderate, and two severe) and only mildly (three cases) or moderately (three cases) evident. In one horse, strands of intermediate signal tissue were observed extending between the APL and straight sesamoidean ligament and were suspected to represent adhesions between these structures.

Desmitis involving both medial and lateral paired PL‐PIPJ was present in 23 out of the 40 injured limbs (58%, CI: 41%–73%). Desmitis involving both medial and lateral paired PL‐PIPJ was evident in 10 limbs with desmitis to the APL (25%, CI: 13%–41%), two limbs with desmitis to the ABPL (5%, CI: 1%–17%), 20 limbs with desmitis of the proximal enthesis of the DDAL (50%, CI: 34%–66%) and one limb with desmitis of the distal enthesis of the PDAL (3%, CI: 0%–13%).

Bilateral changes to the PL‐PIPJ were noted in 11 cases. Out of the 11 bilaterally evaluated cases, 8 (73%, CI: 39%–94%) had been reported to show lameness on the contralateral limb. This was characterised by the presence of a lower lameness grade compared to the dominant lameness prior to diagnostic analgesia or when a switch of the lameness to the contralateral limb was noted after a positive response to diagnostic analgesia. In eight of the 11 cases, the dominant lame limb showed a higher degree of injury to the PL‐PIPJ. Mainly, an additional unilateral desmitis to the APL was noted in the dominant limb in four out of the eight cases. The remaining 18 cases underwent unilateral MR examination.

## 4 DISCUSSION

The first objective of the study was to describe the ability to differentiate the individual PL‐PIPJ. It was determined that reliably differentiating these structures was not always possible. The APL are well distinguishable from the surrounding structures in the majority of cases (81%, CI: 71%–89%); they are located distal to the oblique sesamoidean ligaments and abaxial to the straight sesamoidean ligament, as illustrated in Figure 2. Differentiation between the abaxially located structures was less straightforward (Figure 2), with only 5% (CI: 1%–12%) of the ABPL individually recognisable and 61% (CI: 50%–72%) evident with indistinct margins. The different fibre orientations of both digital annular ligaments might help distinguish them from each other, as well as their anatomic location, as the proximal enthesis of the DDAL ligament is the most abaxially located structure, followed by the distal enthesis of the PDAL located axial to the DDAL and lastly the abaxial palmar/plantar ligament.^2^ Injury to one or more ligaments is subjectively associated with an increased ability to identify the injured structure and differentiate it from surrounding structures. Due to the close proximity of the structures located on the palmar/plantar aspect of the proximal interphalangeal joint, the ability to differentiate between them was not always possible, which may be a result of partial volume averaging. This will interfere with the ability to differentiate structures and subsequently adequately diagnose subtle changes. An increased cross‐sectional area in cases of desmitis will decrease the volume averaging artefact and increase the ability to differentiate structures. Slice thickness in cases of suspected injury to these ligaments should be as thin as possible to minimise the volume averaging artefact.^10^ The authors suggest a slice thickness of 3.5 mm; however, this might not always be practically achievable, as is evident in the range of slice thickness used in this study being T1 GRE (1.6–5 mm), T2 FSE (4–5 mm), STIR (4–5 mm), and T2* GRE (3–5 mm).

The second objective of the study was to describe the characteristics of injury to the PL‐PIPJ. As noted above, the ability to correctly identify injured ligaments may have been impaired by the limitations of low‐field MRI to differentiate between structures, although the increase in cross sectional area seen in injured ligaments may have mitigated this. In addition to cross sectional area, a primary feature of desmitis was altered signal intensity. Signal intensity was mainly graded on T2 FSE images, as on T1 GRE images, magic angle artefact was often evident, as visible in Figure 4. Magic angle artefact has been described for the oblique distal sesamoidean ligaments, to which portions of the PL‐PIPJ have a similar orientation, as shown in Figure 1.^11^, ^12^ Other findings associated with PL‐PIPJ desmitis included the presence of periligamentous oedema and/or enthesophytes. Periligamentous oedema was present only in cases with desmitis of the abaxially located ligaments. A total of 71 abaxially located ligaments were affected with desmitis and periligamentous oedema was present in 75% (*n* = 53/71). The fact that periligamentous oedema was not visible when desmitis of the APL was present is most likely due to the location of the ALP. The APL is bordered by the palmar/plantar pouch of the PIPJ and digital flexor tendon sheath and therefore lack adjacent connective tissue.^2^ As such, these ligaments are less subjected to the formation of periligamentous oedema of surrounding soft tissue structures when injured. Enthesophytes were present in 33% (*n* = 34/102, CI: 24%–43%) of all affected ligaments, but most predominant in cases with desmitis of the proximal enthesis of the DDAL (51%; 30/59 cases). Enthesophytes were best evaluated on the T1W GRE images. Enthesophytes were only evident at the level of the proximal phalanx. Previously, enthesophytes have been commonly diagnosed on ultrasound evaluation at the level of the origin of the ABPL,^6^ which differs from our findings. False negative results on MR examination of other ligaments located in the palmar/plantar pastern have been reported in the literature. Hawkins et al. found that a proportion of cases of distal sesamoidean ligament desmitis diagnosed via ultrasonography were not visible on low‐field MR examination.^12^ Additional high‐field MR examination identified the presence of desmitis in one case.^12^ High‐field MR examination has been reported to be more sensitive compared to ultrasound evaluation.^13^ This could imply that desmitis of the PL‐PIPJ can be overlooked on low‐field MR examination making the incidence of pathology potentially higher than reported here. The sensitivity of imaging modalities for diagnosis of desmitis of these ligaments has not been compared in the literature. The presence of adhesions to adjacent structures was part of the MR assessment. However, due to the size of the ligaments, close relation between structures as shown in Figures 1 and 2 and the difficulty to individually differentiate the abaxially located ligaments as shown in our first objective, evaluation of adhesions was not possible in the abaxially located ligaments.

The third objective was to describe any relationships between the pathology of the PL‐PIPJ, clinical features, and lameness. This study is descriptive in nature and therefore no attempt was made to identify statistically significant differences in either the MR or clinical features of horses with presumed primary PL‐PIPJ desmitis and those with additional pathology. Cases with presumed primary PL‐PIPJ desmitis had a median lameness grade of 3/5 (AAEP scale) with a median duration of 5 weeks (range: 1–36 weeks) prior to presentation. This is in agreement with the previous literature on DDAL desmitis, in which a lameness grade of 2–3 out of 5 was noted.^5^ The level of lameness between both groups was similar. Eleven cases had palpation findings reported; most horses did not show abnormalities during palpation. Horses were however not palpated after MR examination and the palpation technique is not standardised between the different clinicians. The sensitivity of the palpation findings in this retrospective study is therefore questionable. The positive findings in some of the cases do warrant palpation of the region if pathology of the PL‐PIPJ is evident on MR examination. The pattern of response to regional and local anaesthesia in the cases with presumed primary PL‐PIPJ desmitis showed a predominant response to the abaxial sesamoid nerve block. In the cases with additional pathology an abolishment of the lameness was seen with a palmar/plantar digital nerve block in two cases. Three cases showed a significant improvement to intra‐articular analgesia of the proximal interphalangeal joint; these cases showed significant changes to either the middle scutum or the collateral ligament of the proximal interphalangeal joint. Additional findings noted in the multifactorial cases were desmitis of the superficial digital flexor tendon branch (*n* = 2), desmitis of the collateral ligament of the proximal interphalangeal joint (*n* = 6), desmitis of the middle scutum (*n* = 2), navicular bone disease (*n* = 2), desmitis of the collateral ligament of the distal interphalangeal joint (*n* = 1), manica flexoria tear (*n* = 1), or metacarpal subchondral bone injury (*n* = 1). Results noteworthy to mention are the difference in occurrence of severe increase in signal intensity, which was evident in seven cases with presumed primary PL‐PIPJ desmitis, compared to two cases with additional pathology. A severe increase in cross‐sectional area was evident in 12 cases with presumed primary PL‐PIPJ desmitis and in three cases with additional pathology. Finally, severe periligamentous oedema was evident in four cases with presumed primary PL‐PIPJ desmitis and two cases with additional pathology.

The limitations of this study were primarily associated with its retrospective nature. The major limitation of this study is the absence of an unblinded assessment of the MR examinations, which could result in investigator bias. Since the study was conducted in client‐owned horses, no postmortem gross pathology assessment and histopathology were performed to confirm the pathology of the involved ligaments. No confirmation of findings on high‐field MR examination was obtained. There were differences in the MR protocols which influence the uniformity of data; however, the MR data used in the study was deemed of good diagnostic quality. The inability to reliably distinguish the ligaments precludes the full assessment of the ligaments. Finally, there was no uniformity in the lameness assessment and diagnostic analgesia protocol, which makes determining the clinical relevance challenging. Nevertheless, this study has described the clinical features and low‐field MRI findings associated with injury to the PL‐PIPJ, which have not previously been described and therefore provides a valuable basis for future research in this area. A prospective study with a standardised diagnostic analgesia protocol and palpation findings would help to determine the significance of the findings and help establish more specific clinical features, as for example the response to palpation. The low incidence of cases with pathology to these ligaments would make a prospective study challenging.

## 5 CONCLUSION

Thirteen cases were found to have lameness primarily caused by desmitis of the PL‐PIPJ identified on low‐field MR examination; these ligaments should therefore not be overlooked. Three out of four cases had periligamentous oedema when desmitis of the ABPL, proximal enthesis of the DDAL, and/or distal enthesis of the PDAL was present. The presence of periligamentous oedema in the palmar/plantar pastern region on MR evaluation should prompt closer assessment of these ligaments. The PL‐PIPJ is susceptible to magic angle artefact on the T1W GRE sequence. The diagnosis of desmitis should therefore be made on T2W FSE sequence, and both an increase in cross‐sectional area as well as an increase in signal intensity should be evident.

## FUNDING INFORMATION

No funding received.

## CONFLICT OF INTEREST STATEMENT

The authors declare no conflicts of interest.

## AUTHOR CONTRIBUTIONS

**Manon W. J. Peeters:** Conceptualization; investigation; writing – original draft; writing – review and editing; formal analysis; supervision. **Stephan Ott:** Investigation; writing – review and editing. **Elisabeth van Veggel:** Investigation; writing – review and editing. **Dagmar Berner:** Supervision; writing – review and editing; writing – original draft; investigation; conceptualization; formal analysis. **Melanie Perrier:** Supervision; conceptualization; investigation; writing – original draft; writing – review and editing.

## DATA INTEGRITY STATEMENT

Manon Peeters and Dagmar Berner had full access to all the data in the study and take responsibility for the integrity of the data and the accuracy of data analysis.

## ETHICAL ANIMAL RESEARCH

Ethical approval granted via the Clinical Research Ethical Review Board and Social Science Research Ethical Review Board of the Royal Veterinary College.

## INFORMED CONSENT

Explicit owner consent for inclusion of animals in this study was not obtained. Owners/trainers were made aware that case information may be used for research in general.

## PEER REVIEW

The peer review history for this article is available at https://www.webofscience.com/api/gateway/wos/peer-review/10.1111/evj.14547.

## ANTIMICROBIAL STEWARDSHIP POLICY

Not applicable.

## Data Availability

The data that support the findings of this study are openly available in figshare at https://doi.org/10.6084/m9.figshare.29264705.v1, reference number 29264705.
